# Density of Patient-Sharing Networks: Impact on the Value of Parkinson Care

**DOI:** 10.34172/ijhpm.2021.15

**Published:** 2021-03-03

**Authors:** Floris P. Vlaanderen, Yvonne de Man, Marit A. C. Tanke, Marten Munneke, Femke Atsma, Marjan J. Meinders, Patrick P. T. Jeurissen, Bastiaan R. Bloem, Jesse H. Krijthe, Stef Groenewoud

**Affiliations:** ^1^Radboud University Medical Center, Radboud Institute for Health Sciences, Scientific Institute for Quality of Healthcare, Nijmegen, The Netherlands.; ^2^Donders Institute for Brain, Cognition and Behaviour, Nijmegen, The Netherlands.; ^3^Department of Neurology, Radboud Institute for Health Sciences, Nijmegen, The Netherlands.; ^4^Center of Expertise for Parkinson & Movement Disorders, Nijmegen, The Netherlands.; ^5^Department of Intelligent Systems, Delft University of Technology, Delft, The Netherlands.

**Keywords:** Patient-Sharing Networks, Density, Parkinson’s Disease

## Abstract

**Background:** Optimal care for Parkinson’s disease (PD) requires coordination and collaboration between providers within a complex care network. Individual patients have personalised networks of their own providers, creating a unique informal network of providers who treat (‘share’) the same patient. These ‘patient-sharing networks’ differ in density, ie, the number of identical patients they share. Denser patient-sharing networks might reflect better care provision, since providers who share many patients might have made efforts to improve their mutual care delivery. We evaluated whether the density of these patient-sharing networks affects patient outcomes and costs.

**Methods:** We analysed medical claims data from all PD patients in the Netherlands between 2012 and 2016. We focused on seven professional disciplines that are commonly involved in Parkinson care. We calculated for each patient the density score: the average number of patients that each patient’s providers shared. Density scores could range from 1.00 (which might reflect poor collaboration) to 83.00 (which might reflect better collaboration). This score was also calculated at the hospital level by averaging the scores for all patients belonging to a specific hospital. Using logistic and linear regression analyses we estimated the relationship between density scores and health outcomes, healthcare utilization, and healthcare costs.

**Results:** The average density score varied considerably (average 6.7, SD 8.2). Adjusted for confounders, higher density scores were associated with a lower risk of PD-related complications (odds ratio [OR]: 0.901; *P*<.001) and with lower healthcare costs (coefficients: -0.018, *P*=.005). Higher density scores were associated with more frequent involvement of neurologists (coefficient 0.068), physiotherapists (coefficient 0.052) and occupational therapists (coefficient 0.048) (*P* values all <.001).

**Conclusion:** Patient sharing networks showed large variations in density, which appears unwanted as denser networks are associated with better outcomes and lower costs.

## Background

**Key Messages**

** Implications for policy makers**

- Consider to invest in density of patient sharing networks: concentrate care among few (specialised) providers who treat many mutual patients. A high density score can lead to a higher caseload per provider and therefore to more expertise. Our findings suggest that it leads to better outcomes and lower costs.
- Density scores could be used as a quality measure for network organizations.
- Density scores might act as a new tool for research on medical practice variation, or as an aid for contracting strategies of healthcare insurance companies.
- Organizations like ParkinsonNet can stimulate density scores. Enhancing such organizations might contribute to better and affordable care delivery.

** Implications for the public**

Our findings suggest that investing in the density of patient-sharing networks has the potential to increase the value of care and diminish medical practice variation. Higher density is associated with better outcomes and lower costs for patients with PD. Further research might assess if this can also be extrapolated to other chronic conditions.

Achieving optimal care for patients with a chronic neurological condition is challenging.^1^ Optimal management requires a multidisciplinary approach, a complex array of treatment options and a long follow-up. Networks of healthcare providers have proven to be useful for improving coordination and organization of care.^2^ Interestingly, healthcare consists of more than such formal professional networks, because individual patients also build their own informal personalised networks: they choose (or are being allocated to) their own set of healthcare providers, leading to a unique network of providers who treat (‘share’) the same patient.^3^ These so called ‘patient-sharing networks’ of healthcare providers will typically differ in ‘density,’ ie, in the number of identical patients they share.^4^

 Denser patient-sharing networks, ie, networks of providers who share relatively more patients with each other, might result in better care provision. Providers in a dense network might communicate and cooperate better,^5,6^ or know each other through referrals.^6^ This could improve the coordination and organization of care for their patients.^4^ Increased patient-sharing within group practices has been positively associated with patient-reported care coordination.^7^ Positive effects of dense networks might be expected especially among patients with chronic conditions, since these patients likely benefit most from integrated, well-organized care delivery.^1^

 In this study, we aimed to study the effects of network density in the context of a chronic neurological condition, using Parkinson’s disease (PD) as an illustrative example. The care for PD patients is complex, because many different healthcare providers are involved, many of whom work in different echelons of healthcare (primary care, hospitals, long term care).^8,9^ Most persons with PD visit neurologists, physiotherapists, occupational therapists and speech & language therapists. Dieticians and psychologists are also frequently involved, and in advanced PD the number of involved disciplines can be as high as 18.^8^ This provides great challenges to the coordination and organization of multidisciplinary care. Organising care delivery in professional networks of specifically trained healthcare providers at a regional level leads to better collaboration and fewer disease complications,^10^ but there is no evidence that the density of patient-sharing networks improves care delivery and leads to better outcomes. In this study, we therefore assessed the relation between the density of patient-sharing networks and health outcomes, healthcare utilization and healthcare costsfor patients with PD. Specifically, we aimed to investigate (*a*) to what extent patient-sharing networks in PD vary in density in current daily clinical practice (assuming that large variations are generally unwanted); and (*b*) if denser patient-sharing networks are associated with better health outcomes, lower healthcare utilization and lower healthcare costs.

## Methods

###  Data

 We analysed medical claims data from all PD patients in the Netherlands between 2012 and 2016. These data were made available through Vektis, a not-for-profit organization that collects all claims data for all Dutch healthcare insurance companies.^11^ All Dutch inhabitants are obliged by law to have a private healthcare insurance, which is partially paid for by the government. Insurance companies are obliged to accept everybody (against the same price), and the compliance among Dutch citizens to this health insurance obligations is as high as 99.8%.^12^ The database of Vektis therefore contains the claims data of 17.4 million people.^13^ These claims data concern all primary and secondary care, plus the costs for nursing home residency. The Vektis data also include the date when a person died. We successfully used this same Vektis database in a previous analysis where we demonstrated the added value of professional networks of physiotherapists who were specifically trained to treat patients with PD.^14^

###  Study Sample

 We included all 48 769 Dutch insured citizens who had at least one diagnostic related group code (DRG code) of PD since January 2008. This selection was part of the preparation of our database and was performed by Vektis. Data of individual patients were included in the analyses from the moment that the first PD DRG appeared for that patient. The first PD DRG defines the moment of diagnosis by a neurologist. The same approach was used in earlier research on PD care in the Netherlands.^14,15^ The included patients were given a unique random identifier by Vektis. The key to the identifier was not available to the researchers.

 Similar to previous research on claims data for PD care,^15^ we included the PD-related claims data of neurologists, specialized PD nurses (both included at the hospital level, since some hospitals tend to wrongly claim on just one neurologist or specialised nurse while care is provided by many), physiotherapists, occupational therapists, speech & language therapists, dieticians, and psychologists. These are the healthcare providers that are most frequently involved in PD management.^8,10,16-18^ Every healthcare provider was given a random identifier in a similar way as the patients.

###  The Definition of Density

**Figure 1:**
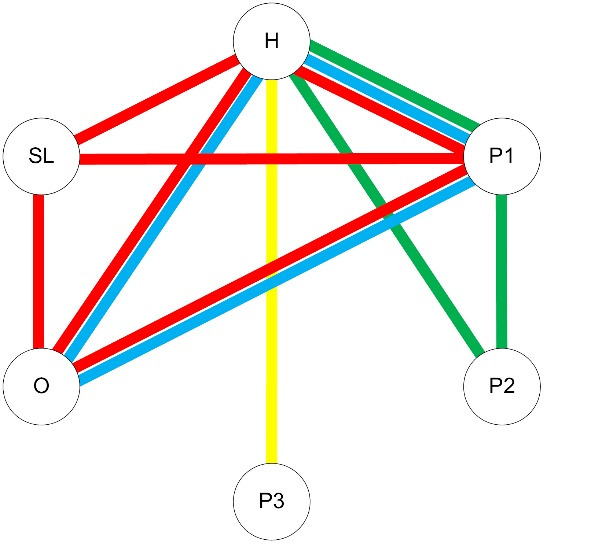
Visual Example for a Situation in Which 6 Healthcare Providers (hospital H, Speech & Language Therapist SL, occupational therapist O, and physiotherapists P1, P2 and P3) Share 4 Patients (Blue, Red, Yellow and Green). The density score of the provider network of the blue patient = (2+2+3)/(3*(2-1)/2) = 2.33, while density scores are 1.67, 1.67, and 1.00 for the red, green, and yellow patient, respectively.

 In order to assess how ‘dense’ a patient-sharing network is, we used the model for care density defined by Pollack et al^4^:


$$density\;score=\frac{\sum_{i=1}^{m}W_{p,i}}{n_{p}\left(n_{p}-1\right)/2}$$

where *n*_p_ is the number of distinct healthcare providers that patient *p* saw, *m* is the total number of possible pairs of these healthcare providers, and w_p,i_ is the number of shared patients for each pair of healthcare providers. The numerator is the total number of instances of patient sharing over the study period among a patient’s providers. The denominator is the total number of pairs of healthcare providers for that patient. The higher the density score, the more patients the involved providers share. A visual example of this method is given in Figure 1. More details of this method can be found in Pollack et al.^4^

###  Comparing Density Scores

 First, the density score per patient was calculated for 36 639 patients. Density scores ranged from 1.00 (which might reflect poor collaboration) to 83.00 (which might reflect better collaboration). For the remaining 12 130 patients it was impossible to calculate a density score because they visited either zero, just one or, due to missing values in the dataset, an unknown number of healthcare providers (Table 1).

**Table 1:** General Characteristics of the Study Sample (n = 48 769)

**Characteristics**		
Average age (in 2012)		71.7 years (SD: 10.1)
Gender		58.9 % men
Time since diagnosis^a^ (y) median (IQR)		5.5 (4.5–7.7)
Follow-up time (y), mean (SD)		3.2 (1.7)
Number (%) of patients with complete follow-up		16 404 (33.6)
Number (%) of patients with less than 6 months follow-up		3544 (7.3)
Number (%) of providers per patient during follow-up	0 or unknown	2222 (4.6)
	1	9908 (20.3)
	>1	36 639 (75.1)
	Median (IQR)	3.0 (2.0–5.0)
Number (%) of patients visiting	Hospital (neurologist/specialized nurse)	40 980 (84.0)
	Physiotherapist	34 496 (70.7)
	Occupational therapist	14 944 (30.6)
	Speech and language therapist	9052 (18.6)
	Dietician	6600 (13.5)
	Psychologist	7549 (15.5)
Number of included	Hospitals	136
	Physiotherapists	14 743
	Occupational therapists	1232
	Speech and language therapists	984
	Dieticians	1286
	Psychologists	238
PD-related healthcare costs per year per patient		
Costs of complications excluded, mean (SD)		$200 499 (3083.59)^b^
Costs of complications included, mean (SD)		$215 347 (3145.99)^b^

Abbreviations: SD, standard deviation; IQR, interquartile range; PD, Parkinson’s disease. ^a^ Calculated to date of death or up to December 31, 2016 (the last day of the dataset); ^b^Euro-dollar conversion per March 13, 2019.

 We then calculated the average density score per hospital across their entire PD patient population, to see if average density scores varied between hospital populations. We therefore assigned all PD patients to the hospital from which they had received most of their hospital care, ie, from which they had the most neurologist and specialized PD nurse claims. We were only able to calculate the average scores for 108 out of all 136 Dutch hospitals, since 28 hospitals did not have any PD patients assigned to them. These 28 hospitals were probably hospitals which fused shortly after the start of our time span with other hospitals, or were large specialised medical clinics which do not treat PD.

 To visualize the differences in density scores, we selected the 3 hospital populations with the highest and 3 other hospital populations with the lowest density score. The average density score per hospital population was positively influenced by the number of assigned patients. Regarding the lowest density scores, we therefore only considered hospital populations with at least 369 patients, which was the size of smallest hospital population with the highest density score. We visualized the networks of the selected hospital populations by plotting the healthcare providers that shared mutual patients with t-distributed stochastic neighbour embedding (t-SNE).^19^

###  Outcomes, Utilization and Costs

 Our next main aim was to assess the relation between density scores and health outcomes, healthcare utilization and healthcare costs. For this purpose, we defined the following outcome measures. *Health outcomes* were defined as the occurrence of any one or more of 3 PD-related complications (ie, a claimed DRG for pneumonia, orthopaedic injury or hospital admission for PD)^14,15^ and mortality. *Healthcare utilization* was defined as the number of DRGs (neurologist and specialized PD-nurses) or visits (allied healthcare providers) to the included healthcare providers. *Healthcare costs* were defined as the sum of the prices of the claims. We calculated healthcare costs separately with and without the costs of the DRGs of PD-related complications.

 Subsequently, we used regression analyses for each outcome. To assess the association between density scores and health outcomes we used logistic regression models since the dependent variables were all dichotomous; the associations with utilization and costs were performed with linear regression models. In the linear regression analyses we log-transformed the dependent variables since the effects on utilization and costs were multiplicative rather than additive and the residuals were closer to the normal distribution if the dependent variables were log-transformed. For similar reasons, and to unify our results and simplify the interpretation, we log-transformed the density scores in all regression analyses.

 In all regression analyses, we adjusted for age, sex, the duration of the disease, the number of healthcare providers per patient, the average number of patients per healthcare provider in the patient’s network, and the follow-up time. Age, sex and the duration of the disease might influence the dependent variables, and these were therefore added to the regression model as independent variables. For duration of the disease, we added an extra variable indicating if a patient had PD-related claims in 2008 or not. This was done to cope with the limitation that our dataset does not contain data prior to 2008, which would otherwise result in underestimation of disease duration. The number of healthcare providers per patient and the average number of patients per provider in the patient’s network were added in a similar way, since these variables appeared to have a correlation with density of -0.093 and 0.254 respectively (*P* values both <.001).

 For all regression models, we excluded patients with either zero, or one or an unknown number of healthcare providers. In the logistic regression models on PD-related complications, we additionally excluded patients of which we did not have the full follow-up time available (5 years), since these patients would have had less time to develop a complication. This resulted in 13 129 included patients. In the linear regression models, we adjusted for follow-up by defining the dependent variables as averages per month. Patients with less than 6 months follow-up were excluded to avoid outliers. This resulted in 35 414 included patients for the linear regression analyses on healthcare costs. For the linear regression analyses on utilization, only patients could be included that had claims from the designated healthcare providers. We included 33 703 for neurology utilization, 33 474 for physiotherapist utilization, and 14 534 for occupational therapist utilization. For speech and language specialist utilization 8895 patients could be included, and the numbers for dieticians and psychologist utilization were 6490 and 6437, respectively.

###  Secondary Analysis

 Our results might be influenced by the activities of the Dutch nationwide ParkinsonNet healthcare network. Covering the entire country, ParkinsonNet is a Dutch not-for-profit organization, supporting regional provider networks of medical and allied healthcare professionals specialized in the management of patients with PD. The ParkinsonNet approach stimulates concentration of care among the specifically trained professionals (which influences density scores), but also develops guidelines, stimulates collaboration and provides education to healthcare providers.^10,20^ These efforts have led to improved health outcomes, and decreased healthcare costs.^10^

 To assess the relation between the density score and membership of the ParkinsonNet network, we identified for all included providers if they were a ParkinsonNet member or not. Since the claims of neurologists and specialized PD-nurses in our data set were only available at the hospital level, and because membership of ParkinsonNet is individual, we excluded the neurology DRGs from this analysis. Subsequently, we performed an additional linear regression analysis on the log-transformed density scores to identify if there was a correlation between the log-transformed density score and the percentage of visits to ParkinsonNet healthcare providers. The variables ‘number of healthcare providers per patient’ and ‘the average number of patients per provider in the patient’s network’ were added as independent variables, since they influence the dependent variable (log-transformed density score). After exclusion of the hospital claims and exclusion of patients with (then) zero, one or an unknown number of healthcare providers, 36 639 patients could be included in this linear regression model.

###  Data Availability Statement

 The data that support the findings of this study are available from the corresponding author, upon reasonable request. However, the original claims data belong to Vektis. Permission of Vektis is required before original claims data can be made available due to privacy laws.

## Results

###  Variation in Density 

**Figure 2:**
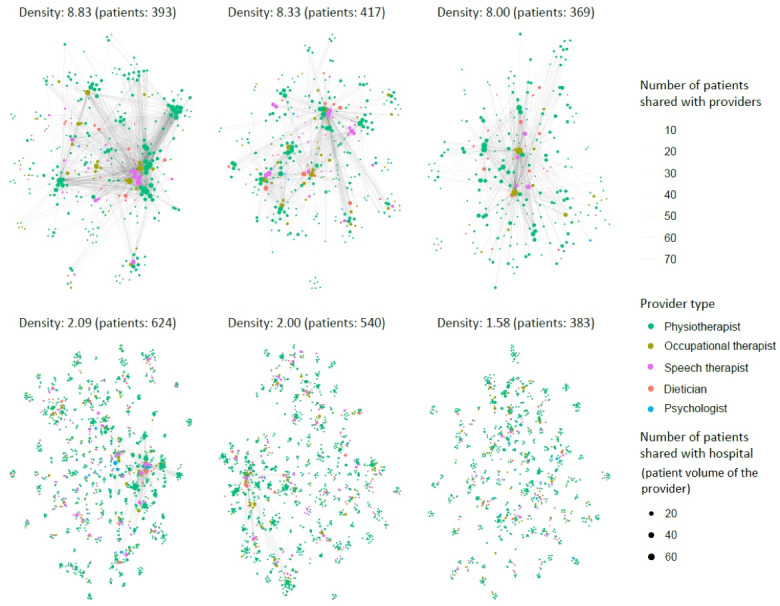
t-SNE Visualization of Top 3 and Bottom 3 Hospital Populations in Terms of Average Density Scores. Abbreviation: t-SNA, t-distributed stochastic neighbour embedding.

 The characteristics of the study sample are presented in Table 1. The average density score varied considerably. At the individual patient level, the average score was 6.7 (SD: 8.2). At the level of hospital populations, it was 3.9 (SD: 1.8). This difference in average scores arose because many hospitals had very few patients assigned (17 hospitals had less than 100 patients). The t-SNE visualization of the variation between hospital populations is shown in Figure 2. For the top 3 hospital populations, providers shared more patients with the hospital (bigger dots) and also with each other (more clusters, more darker lines).

 Each dot represents a provider; the colour represents provider type; the size of the dot represents the number of shared patients with the hospital (the hospital itself is not shown); lines and relative location between the dots represent the number shared patients with each other (only when 10+ patients are shared, a line is shown); when more patients are shared, the line is darker.

###  Regression Models on Health Outcomes, Utilization and Costs 

Table 2 shows that higher density scores were associated with lower incidences of PD-related complications (OR: 0.901, *P* value <.001). A doubling of the density score was associated with lower odds of complications of approximately 1-2^log(0.901) ≈ 7.0%. In a similar way, denser patient-sharing networks were associated with a lower occurrence of pneumonias and orthopaedic injuries, but not with lower PD-related hospitalization.

**Table 2:** Adjusted Estimates Relating Log(Density Score) to Health Outcomes, Healthcare Utilization and Healthcare Costs

**Health Outcomes**	**OR**	**95% CI**	***P*** ** Value**
Incidence of PD-related complications			
Incidence of pneumonia	0.926	0·889–0·964	<.001
Incidence of orthopedic injuries	0.899	0·864–0·936	<.001
Incidence of PD-related hospitalizations	1.023	0·971–1·079	.392
Incidence of all PD-related complications	0.901	0·862–0·941	<.001
Mortality	0.962	0·926–1·000	.052
**Healthcare Utilization **	**Coefficients **	**95% CI**	***P*** ** Value**
Log-transformed neurologist utilization	0.068	0·062–0·075	<.001
Log-transformed physiotherapist utilization	0.052	0·038–0·065	<.001
Log-transformed occupational utilization	0.048	0·028–0·068	<.001
Log-transformed speech and language therapist utilization	0·024	-0·009–0·057	.156
Log-transformed dietician utilization	-0.013	-0·043–0·017	.409
Log-transformed psychology utilization	-0.032	-0·061 – -0·003	.029
**Healthcare Costs**	**Coefficients **	**95% CI**	***P*** ** Value**
Log-transformed costs excluding claims for complications	-0.018	-0·031 – -0·006	.005
Log-transformed costs including claims for complications	-0.030	-0·043 – -0·018	<.001

Abbreviations: PD, Parkinson’s disease; OR, odds ratio. All values are adjusted for the effects of age, sex, the duration of the disease, the number of healthcare providers per patient, the average number of patients per healthcare provider in the patient’s network and the differences in follow-up time.

 Higher density scores were associated with more frequent involvement of neurologists, physiotherapists and occupational therapists (coefficients of 0.068, 0.052 and 0.048, respectively, *P* values all <.001), but to less frequent involvement of psychologists (coefficient of -0.032; *P* =.029). A doubling of the density score was associated with an increase 4.8% for neurologist utilization, 3.7% for physiotherapist utilization, and 3.4% for occupational therapist utilization. Similarly, an exponential increase in density score was associated with decreased psychologist utilization of approximately 2.2%. For speech & language therapists and dieticians we found no associations.

 Healthcare costs seemed to be negatively associated with density scores (Table 2). A doubling of the density score was associated with reduced healthcare costs of approximately 1.2% (complications excluded) to 2.1% (complications included). Compared to the average healthcare costs for PD (Table 1), this would equate to an annual reduction of $2406 to $4522 per patient (over 36 639 patients, this corresponds with $0.9 million to $1.7 million). Supplementary file 1 shows the complete regression analyses on health outcomes (Table S1), healthcare utilization (Table S2), and healthcare costs (Table S3).

###  Secondary Analysis

 A high percentage of visits to ParkinsonNet members was associated with higher density scores (coefficient of 1.164; *P* < .001). Patients who exclusively consulted ParkinsonNet professionals had an approximately exp(1164) ≈ 3.2 times higher density score compared to patients who never consulted ParkinsonNet members. The linear regression analysis is included as Supplementary file 1 (Table S4).

## Discussion

 Our aim was to identify whether provider networks for individual PD patients differ in density and, if so, whether denser patient-sharing networks would be associated with higher value of care. These questions were addressed in a unique cohort of all PD patients in the Netherlands followed over a 5-year timeframe. Several findings emerged. First, we identified substantial differences in the density scores between patient-sharing networks. These differences were found both at the level of the individual patient and at the level of hospital populations. Second, our analyses show that denser patient-sharing networks are associated with a lower occurrence of PD-related complications, especially fewer pneumonias and orthopaedic injuries. Third, denser patient-sharing networks are associated with more common involvement of neurologists, physiotherapists and occupational therapist services, but also with somewhat lower utilization of psychologists. Fourth, denser patient sharing networks are associated with a small decrease in healthcare costs for PD management. Finally, our secondary analysis shows a strong correlation between the density score and the percentage of visits to providers associated with ParkinsonNet, a Dutch network of specialised healthcare professionals. This suggests that the observed effects might be influenced by the efforts of this integrated network approach.

 Comparison of our study to previous studies is difficult, since no prior work assessed patient-sharing network densities nor their effects in a PD context. Two large studies about the density of patient-sharing networks in the United States showed no impact on quality of care, while healthcare utilization and costs increased.^21,22^ Both studies focused on the densities of general Medicare insured patients, rather than on patients with chronic conditions for whom the bests results can be expected. When focusing on patients with chronic conditions, the average density values that we identified at the individual patient level are comparable with density values of patient-sharing networks of patients with diabetes, congestive heart failure, chronic obstructive pulmonary disease and cancer.^4,23,24^ This supports the validity of our approach. Research on these other chronic conditions identified similar associations between care density and health outcomes: cancer survivors with denser networks are hospitalized less often,^23^ while diabetes patients with denser networks have a lower risk of being readmitted to hospital and to experience potentially avoidable complications.^24^ The last study found no positive associations in the context of congestive health failure and chronic obstructive pulmonary disease, and even found negative associations with some other quality measures. Additionally, denser patient-sharing networks have been linked to reduced healthcare costs for patients with congestive heart failure,^4^ diabetes,^4^ and cancer.^23^

 Our method has several strengths. First, we followed a clear and predefined set of analyses. Second, our dataset contained all diagnosed patients in the country, which limits the risk of selection bias. However, some selection bias might have been introduced because inclusion was based on the PD DRG. Our sample may have included cases for whom the initial diagnosis of PD turned out to be incorrect. PD can be difficult to diagnose in early disease stages, with reported diagnostic error rates of >10%,^25^ eg, because forms of atypical parkinsonism can present like PD.^26^ Because our sample matches well with the population characteristics of other studies in terms of prevalence,^26^ age,^14,26^ division of sex,^14,26,27^ and healthcare use,^14^ we do not think this has greatly affected our findings. And more importantly, a certain rate of misdiagnosis is a reality in daily clinical practice, so our findings are pragmatic in the sense that they apply to a real-life population of patients with both PD and parkinsonism. Another strength is that our study was based on highly standardized claims data. These data are a fair representation of the care provided, even though there is not a 100% match: mis-registrations or non-registrations can occur, as well as errors during the process of data registration by a healthcare professional or during the transfer of hospital data to the current database. A further strength is that we had a rather long follow-up relative to previous studies with a comparable approach. Finally, by using regression models, we did not have to divide our study sample into groups of lower or higher density values. This way, we avoided loss of data, making the analyses more accurate.

 Our study also had some limitations, some of which relate to the generic limitations of observational studies of large data sets.^28^ For example, for primary care providers we were unable to determine if all claims were PD-specific, and their inclusion could lead to an overestimation of utilization and costs. Second, it was technically impossible to assess the duration of the disease when a patient had received the diagnosis before January 1, 2008, which might have influenced our estimates for disease duration. A third limitation is that we faced many (arbitrary) decisions when defining our methods. There are other models for assessing care density, for example, the model of Landon.^3^ We preferred the method of Pollack et al since it uses the patient’s perspective, rather than the provider’s perspective.^4^ Another methodological decision included the assignment of patients to a hospital, but not to other providers. For all such decisions, we tried to choose methods that had been used in previous research. However, given the limited PD-specific literature available for this topic, this was not always possible. A methodologic limitation is that not all providers in PD are always involved. When a less frequently involved provider is included in the patient’s network, its density score will likely drop. However, this effect is partially mitigated by the inclusion of the independent variable “number of providers per patient”, since less frequently involved providers will usually be involved in advanced stage of the disease when the more frequently involved providers are already present. Another limitation is that we did not analyze the characteristics of patients who were excluded from the analyses. We regarded them as data errors, or they might have received the diagnosis shortly before the end of the study period or they might have died just after the start of the study period. A final limitation is that our analyses did not adjust for correlations within hospital populations. However, we expect these correlations to be only small. This was corroborated by repeating the analyses using generalized estimating equations, which resulted in small estimated correlations between the residuals of patients within a hospital and little effect on the estimated coefficients in the models.

 Our findings suggest that investing in the density of patient-sharing networks has the potential to increase the value of care for individual patients with PD. For individual healthcare providers, investing in density can be achieved by increasing the caseload of unique patients with PD, which in turn might lead to greater expertise. At the level of the patient population, this increased expertise might lead to better value of care and less medical practice variation. Such variations in care delivery are frequently reported in PD,^29-31^ but these are obviously unwanted as it leads to inequality in access to good care for different patients. And more importantly, a higher density was associated with better value of care and with better outcomes and lower costs. We therefore recommend that density scores be considered as a quality measure for network organizations. At the level of societies, density scores might act as a new tool for research on medical practice variation, or as an aid for contracting strategies of healthcare insurance companies. Further research might assess if this only applies to PD, or whether such an approach can also be extrapolated to other chronic neurological conditions.

## Ethical issues

 This study was approved by the institutional review board of the Radboud University Medical Centre with a waiver of consent for participants in the study (file number 2019-5106).

## Competing interests

 MACT reports that she worked for VGZ (healthcare insurer) during the conduct of this study. MJM reports grants from Health Holland, Top Sector Life Sciences & Health, during the conduct of the study. BRB reports grants from Netherlands Organization for Health Research and Development, during the conduct of the study; personal fees from serving on the scientific advisory boards of Abbvie, Biogen, UCB, and Walk with Path, personal fees from speaking at a conference for Abbvie, Zambon, and Roche and Bial, grants from the Netherlands Organization for Scientific Research, the Michael J Fox Foundation, UCB, Abbvie, the Stichting Parkinson Fonds, the Parkinson’s Foundation, Verily Life Sciences, Horizon 2020, the Topsector Life Sciences and Health, and the Parkinson Vereniging, outside the submitted work. JHK reports grants from ZonMW, during the conduct of the study.

## Authors’ contributions

 FPV contributed by acquisition of data, analysis and interpretation of data, drafting the manuscript and statistical analysis. YdM and JHK contributed by acquisition of data, analysis and interpretation of data and statistical analysis. MACT and MM contributed by conception and design and administrative, technical or material support. FA contributed by statistical analysis and administrative, technical or material support. MJM, PPTJ, and BRB contributed by critical revision of the manuscript and supervision. SG contributed by conception and design, critical revision of the manuscript, obtaining funding, and supervision.

## Funding

 This study was funded by The Netherlands Organization for Health Research and Development (ZonMw, project number 91215076), the Celsus Academy for sustainable Healthcare, and the Ministry of Economic Affairs by means of the PPP Allowance (grant LSHM16009) made available by the Top Sector Life Sciences & Health to stimulate public-private partnerships.

 The funding sources of this study had no involvement in the study design; in the collection, analysis, and interpretation of data; in the writing of the report; nor in the decision to submit the paper for publication. The corresponding author had full access to all the data in the study and had final responsibility for the decision to submit for publication.

## Supplementary files

Supplementary file 1 contains Tables S1- S4.
