# Supplementary material for: Density of Patient-Sharing Networks: Impact on the Value of Parkinson Care
Source: Int J Health Policy Manag. 2021 Mar 3;11(7):1132–9. doi: 10.34172/ijhpm.2021.15 (PMC9808175; doi:10.34172/ijhpm.2021.15)
Supplement: Supplementary file 1 — contains Tables S1- S4. [file ijhpm-11-1132-s001.pdf]

Supplementary file 1

Table S1. Odds ratios for logistic regression on health outcomes (adjusted)															
	Pneumonia			Orthopedic injuries			PD-related hospitalization			All PD-related complications			Mortality		
**Parameter**	**Estim.**	**95% C.I.**	**P-value**	**Estim.**	**95% C.I.**	**P-value**	**Estim.**	**95% C.I.**	**P-value**	**Estim.**	**95% C.I.**	**P-value**	**Estim.**	**95% C.I.**	**P-value**
**(intercept)**	0·113	0·083 - 0·154	<0·001	0·418	0·309 - 0·566	<0·001	0·666	0·456 - 0·973	0·036	0·778	0·556 - 1·090	0·144	0·000	0·000 - 0·000	<0·001
**log(Density score)**	0·926	0·889 - 0·964	<0·001	0·899	0·864 - 0·936	<0·001	1·023	0·971 - 1·079	0·392	0·901	0·862 - 0·941	<0·001	0·962	0·926 - 1·000	0·052
**Age**	1·028	1·024 - 1·032	<0·001	1·014	1·010 - 1·018	<0·001	0·964	0·960 - 0·969	<0·001	1·015	1·011 - 1·019	<0·001	1·129	1·124 - 1·135	<0·001
**Sex (woman)**	1·134	1·059 - 1·215	<0·001	1·520	1·417 - 1·630	<0·001	0·969	0·887 - 1·059	0·491	1·404	1·296 - 1·522	<0·001	0·665	0·621 - 0·713	<0·001
**Duration of PD**	0·971	0·921 - 1·023	0·271	1·063	1·009 - 1·120	0·022	1·421	1·318 - 1·533	<0·001	1·072	1·011 - 1·136	0·019	1·136	1·076 - 1·198	<0·001
**PD in 2008**	1·168	1·028 - 1·327	0·017	0·988	0·870 - 1·123	0·858	1·054	0·892 - 1·248	0·537	1·053	0·911 - 1·215	0·483	1·193	1·051 - 1·354	0·007
**Number of providers per patient**	1·014	1·000 - 1·027	0·042	1·064	1·049 - 1·080	<0·001	1·080	1·063 - 1·098	<0·001	1·074	1·056 - 1·093	<0·001	0·828	0·815 - 0·841	<0·001
**Number of patients per provider**	0·844	0·809 - 0·881	<0·001	0·931	0·893 - 0·970	0·001	0·983	0·928 - 1·040	0·547	0·888	0·849 - 0·929	<0·001	0·868	0·837 - 0·900	<0·001
**AUC**	0·601			0·606			0·671			0·616			0·788		
**N**	14,361			14,361			14,361			14,361			20,603		

Table S2. Parameter estimates for regression on log-transformed healthcare utilization (adjusted)																		
	**Number of neurologist visits**			**Number of physiotherapist visits**			**Number of occupational therapist visits**			**Number of speech & language therapist visits**			**Number of dietician visits**			**Number of psychologist visits**		
**Parameter**	**Estim.**	**95% C.I.**	**P-value**	**Estim.**	**C.I.**	**P-value**	**Estim.**	**C.I.**	**P-value**	**Estim.**	**C.I.**	**P-value**	**Estim.**	**C.I.**	**P-value**	**Estim.**	**C.I.**	**P-value**
**(intercept)**	1·009	0·958 - 1·061	<0·001	1·150	1·046 - 1·254	<0·001	-0·313	-0·462 - - 0·164	<0·001	0·678	0·450 - 0·906	<0·001	-1·038	-1·232 - - 0·844	<0·001	-0·171	-0·362 - 0·019	0·078
**log(Density score)**	0·068	0·062 - 0·075	<0·001	0·052	0·038 - 0·065	<0·001	0·048	0·028 - 0·068	<0·001	0·024	-0·009 - 0·057	0·156	-0·013	-0·043 - 0·017	0·409	-0·032	-0·061 - - 0·003	0·029
**Age**	-0·015	-0·015 - - 0·014	<0·001	0·013	0·011 - 0·014	<0·001	0·013	0·011 - 0·014	<0·001	0·002	-0·001 - 0·005	0·143	0·015	0·013 - 0·018	<0·001	-0·004	-0·006 - - 0·001	0·003
**Sex (female)**	-0·045	-0·057 - - 0·033	<0·001	0·036	0·011 - 0·060	0·004	0·070	0·039 - 0·101	<0·001	-0·157	-0·209 - - 0·105	<0·001	0·061	0·017 - 0·106	0·006	0·029	-0·020 - 0·079	0·243
**Duration of PD**	-0·140	-0·143 - - 0·136	<0·001	-0·065	-0·072 - - 0·057	<0·001	-0·115	-0·125 - - 0·105	<0·001	-0·121	-0·137 - - 0·105	<0·001	-0·145	-0·160 - - 0·131	<0·001	-0·115	-0·131 - - 0·100	<0·001
**PD in 2008**	0·370	0·351 - 0·390	<0·001	0·367	0·328 - 0·407	<0·001	0·382	0·333 - 0·431	<0·001	0·399	0·322 - 0·476	<0·001	0·426	0·357 - 0·496	<0·001	0·158	0·079 - 0·237	<0·001
**Number of providers per patient**	0·021	0·018 - 0·024	<0·001	0·179	0·173 - 0·184	<0·001	0·053	0·047 - 0·059	<0·001	0·049	0·039 - 0·058	<0·001	0·020	0·012 - 0·028	<0·001	-0·005	-0·015 - 0·005	0·347
**Number of patients per provider**	-0·010	-0·017 - - 0·003	0·003	-0·175	-0·189 - - 0·161	<0·001	-0·056	-0·078 - - 0·035	<0·001	-0·041	-0·078 - - 0·003	0·032	-0·041	-0·072 - - 0·009	0·012	-0·014	-0·042 - 0·013	0·310
**R^2**	0·248			0·212			0·065			0·040			0·080			0·054		
**N**	33,703			33,474			14,534			8,895			6,490			6,437		

Table S3. Parameters of regression on log-transformed healthcare costs (adjusted)						
	Without costs for PD-related complication costs			With costs for PD-related complication costs		
Parameter	Estim.	C.I.	P-value	Estim.	C.I.	P-value
(intercept)	3·319	3·221 - 3·417	<0·001	3·539	3·443 - 3·635	<0·001
Log(Density score)	-0·018	-0·031 - -0·006	0·005	-0·030	-0·043 - -0·018	<0·001
Age	0·043	0·042 - 0·044	<0·001	0·043	0·041 - 0·044	<0·001
Sex (woman)	0·132	0·109 - 0·155	<0·001	0·133	0·110 - 0·155	<0·001
Duration of PD	0·032	0·025 - 0·039	<0·001	0·036	0·030 - 0·043	<0·001
PD in 2008	0·458	0·421 - 0·496	<0·001	0·413	0·377 - 0·450	<0·001
Number of providers per patient	0·015	0·010 - 0·020	<0·001	0·015	0·010 - 0·020	<0·001
Number of patients per provider	-0·075	-0·087 - -0·062	<0·001	-0·079	-0·092 - -0·067	<0·001
R^2	0·206			0·210		
N	35,414			35,414		

Table S4. Regression on the log-transformed density scores (adjusted)			
Parameter	estimates	95% CI	P-value
(intercept)	0·648	0·620-0·675	<0·001
% ParkinsonNet provider visits	1·164	1·145 – 1·184	<0·001
Number of providers per patient	0·005	0·002-0·009	0·004
Number of patients per provider	0·191	0·183 – 0·199	<0·001
R^2	0·325		
N	36639		
